# Measurements of Wide-Band Cochlear Reflectance in Humans

**DOI:** 10.1007/s10162-012-0336-1

**Published:** 2012-06-12

**Authors:** Daniel M. Rasetshwane, Stephen T. Neely

**Affiliations:** Boys Town National Research Hospital, 555 North 30th Street, Omaha, NE 68131 USA

**Keywords:** otoacoustic emissions, latency, impedance, gammatone

## Abstract

The total sound pressure measured in the ear canal may be decomposed into a forward- and a reverse-propagating component. Most of the reverse-propagating component is due to reflection at the eardrum. However, a measurable contribution to the reverse-propagating component comes from the cochlea. Otoacoustic emissions (OAEs) are associated with this component and have been shown to be important noninvasive probes of cochlear function. Total *ear-canal reflectance* (ECR) is the transfer function between forward and reverse propagating components measured in the ear canal. *Cochlear reflectance* (CR) is the inner-ear contribution to the total ECR, which is the measured OAE normalized by the stimulus. Methods are described for measuring CR with a wide-band noise stimulus. These measurements offer wider bandwidth and minimize the influence of the measurement system while still maintaining features of other OAEs (i.e., frequency- and level-dependent latency). CR magnitude decreases as stimulus level increases. Envelopes of individual band-limited components of the time-domain CR have multiple peaks with latencies that persist across stimulus level, despite a shift in group delay. CR has the potential to infer cochlear function and status, similar to other OAE measurements.

## Introduction

Sound pressure measured in the ear canal may be regarded as being composed of two components: (1) a forward-propagating component that transports acoustic energy into the inner ear for the purpose of hearing and (2) a reverse-propagating component that is produced in response to the forward component. In human ears, much of the reverse-propagating component comes from the eardrum. However, a small but measurable contribution to the reverse-propagating component comes from the cochlea, the primary sensory organ for hearing, which is located within the inner ear. We refer to the transfer function between forward- and reverse-propagating components measured in the ear canal as *ear-canal reflectance* (ECR). We refer to the inner-ear contribution to this reflectance, which is equivalent to a measurement of otoacoustic emission (OAE) normalized by the stimulus, as *cochlear reflectance* (CR). In this study, methods to extract CR from measurements of ECR are described. The CR extraction method includes two steps: (1) subtraction of high-level ECR (with mostly linear cochlear response) from low-level ECR (with both linear and nonlinear responses) and (2) time–frequency analysis. The objective is to obtain a cochlear response that has the least possible influence of the middle-ear and measurement system and that can be interpreted in terms of a linear model. CR measurements may provide alternative means for clinical prediction of supra-threshold cochlear status and for future cochlear modeling interpretations. Abbreviations and symbols used in this article are listed in Table 1.

**TABLE 1:** List of abbreviations and symbols

Abbreviation or symbol	Meaning
ECR	Ear-canal reflectance
CR	Cochlear reflectance
WBN	Wideband noise
LSC	Linear-swept frequency chirp
TF	Transfer function
*L*	Stimulus level
*r*_WBN, *L*_ (*t*)	Time-domain reflectance measured using wideband noise stimulus at stimulus level *L*
*r*_LSC_ (*t*)	Time-domain reflectance measured using linear-swept frequency chirp
*r*_CR,*L*_ (*t*)	Time-domain cochlear reflectance measured at stimulus level *L*
*f*_c_	Center frequency of gammatone filter
ERB (*f*_c_)	Equivalent rectangular bandwidth of gammatone filter with center frequency *f*_c_
*r*_CR, *L*_ (*f*_c_, *t*)	Time-domain cochlear reflectance measured at stimulus level *L* for gammatone filter with center frequency *f*_c_
*N*_*L*_	Lower bound of cochlear reflectance in time–frequency domain
*N*_*H*_	Upper bound of cochlear reflectance in time–frequency domain
*τ*_CR_ (*f*_c_)	Group delay in milliseconds of gammatone filter with center frequency *f*_*c*_
*N*_CR_ (*f*_c_)	Group delay in cycles of the center frequency *f*_c_ of gammatone filter with center frequency *f*_c_
*f*_*a*|*b*_	Break point frequency in group delay
CRM	Cochlear reflectance magnitude
CRM_*L*_ (*f*_c_)	Cochlear reflectance magnitude as a function of center frequency *f*_c_ at stimulus level *L*
CRM_*L*_	Cochlear reflectance magnitude at stimulus level *L*, obtained from CRM_*L*_ (*f*_c_) by summing across frequency

Otoacoustic emissions are acoustical signals that originate within the cochlea as by-products of its normal signal-processing function and constitute the reverse-propagating component of sound pressure that originates from the cochlea. OAEs may be generated either spontaneously, in the absence of any acoustical stimulation, or as a response evoked by stimulation. OAEs are generated by vibrations within the cochlea at various locations. These vibrations travel towards the base of the cochlea, through the middle ear, and to the ear canal where they can be detected as sound pressure. OAEs can be evoked using (1) a transient stimulus, (2) a pure tone, or (3) a pair of tones. The type of stimulus determines the OAE name. Stimuli for transient-evoked OAEs (TEOAE) include clicks (click-evoked OAE or CEOAE) and short-duration tone bursts and evoke a wider frequency range of OAE simultaneously (with clicks evoking a wider frequency range compared to tone bursts). Stimulus-frequency OAEs (SFOAEs) are evoked using pure tones and cover a narrow frequency range around the frequency of the stimulus. Distortion-product OAEs (DPOAEs) are evoked using a pair of primary tones. OAEs have also been evoked using noise with a band-limited spectral density that is similar to that of a click used for evoking CEOAE (Maat et al. 2000).

OAEs can also be classified based on the mechanism or “source” of emission (e.g., Shera and Guinan 1999). It is generally accepted that there are two distinct OAE generation mechanisms—linear coherent reflection and nonlinear distortion (e.g., Shera and Guinan 1999). OAEs produced by these two mechanisms have different properties. For example, the phase of the linear coherent reflection component changes rapidly with frequency, while the phase of the nonlinear distortion component is essentially flat as a function of frequency (e.g., Shera and Guinan 1999; Kemp 2002; Dhar et al. 2011). Although it is generally agreed that linear coherent reflection is the primary mechanism involved in SFOAEs and that nonlinear distortion is the primary mechanism involved in DPOAEs, details of these OAE generation mechanisms are still a matter of debate (e.g., Siegel et al. 2005; Yates and Withnell 1999). Knowledge of the precise mechanism is important because it affects the particular processing and decoding of OAE data used to extract information relevant to the hearing process (Kemp 2002). The generation mechanisms may also correlate with particular cochlear pathologies (Shera and Guinan 1999), although empirical evidence in support of this view remains elusive.

CR is the cochlear contribution to the total ear-canal reflectance measured in the ear canal. It is essentially the measured OAE normalized by the stimulus. Specifically, the OAE waveform is deconvolved by the forward pressure. Our definition of CR should not be confused with Shera et al. (2005) who define CR looking into the cochlea from the stapes. A complete characterization of reflectance, as with any transfer function, requires measurements across a wide frequency range. In linear, time-invariant systems, transfer functions are independent of the stimulus used to measure them. However, CR is stimulus dependent because cochlear nonlinearities are functionally significant. This study measured CR using a wide-band noise (WBN) stimulus over a range of stimulus levels. Use of a WBN stimulus allows for a characterization of the measurements as a transfer function and also offers other benefits as described later. These measurements have the potential to infer cochlea function and status, similar to OAE measurements.

Ear-canal reflectance (ECR) can be measured by placing a microphone and an appropriately calibrated sound source in the ear canal (e.g., Allen 1986; Keefe et al. 1992; Siegel 1994; Neely and Gorga 1998). Most of the reflected energy that constitutes ECR is linearly related to stimulus intensity and comes from the eardrum and middle ear (for a review, see Keefe and Schairer 2011); however, a small contribution, with both linear and nonlinear components, is due to reflection from the cochlea (Allen 1997). Allen described a method for measuring the nonlinear component of the ECR. They measured ECR at several different stimulus levels, using tonal stimuli and a calibrated sound source. By substituting a high-level impedance for the characteristic impedance when calculating reflectance, they extracted a nonlinear component of reflectance. Their results demonstrated that cochlear reflectance varied with stimulus level. However, the interpretation of tonal responses as a transfer function presents a theoretical problem because the interpretation of reflectance as a transfer function assumes a linear, time-invariant system. When the system is nonlinear, then it may no longer be valid to combine independent measurements made one frequency at a time and interpret the set of frequencies as if they represent a single linear system. Use of a WBN stimulus in the current measurements allows for the invocation of de Boer’s (1997) nonlinear equivalence (EQ-NL) theorem, which in turn theoretically allows for the interpretation of the data using concepts and principles applicable to linear systems such as transfer functions, Fourier analysis, and time–frequency analysis. According to the EQ-NL theorem, for a given class of nonlinear system, of which the cochlea is an example, there is an equivalent linear system that has the same response as the nonlinear system for a WBN stimulus at a specific level.

This study describes methods for extracting CR from ECR. Our methods for extraction assume that noncochlear contributions to ECR vary linearly with stimulus level, while CR varies mainly nonlinearly with level. We demonstrate the level dependence of CR measurements and show that CR latency has frequency and level dependence that is a characteristic of signals of cochlea origin. Qualitative comparisons are made to previous measurement of transfer functions (TFs) of CEOAE and SFOAE (Kalluri and Shera 2007; Sisto and Moleti 2008). We apply time–frequency analysis to our estimate of time-domain CR to obtain frequency band-limited estimates that may be useful in probing the function and status of spatially limited regions of the cochlea. We examine envelopes of these band-limited CR envelopes and demonstrate that they have multiple peaks with delays that persist across stimulus level, despite the shift in group delays with level. This analysis may potentially provide more information regarding the generation sites of OAEs.

## Methods

### Subjects

A total of 20 subjects with ages ranging from 15 to 65 years participated in this study. Subjects were required to have audiometric thresholds of 20 dB HL or better (ANSI 1996) for the octave and interoctave frequencies from 0.25 to 8 kHz. Middle-ear status was assessed using tympanometry with a 226-Hz probe tone. To qualify for inclusion, the following tympanometric criteria had to be met: peak-compensated static admittance of 0.3–2.5 mmhos and peak pressure between −100 and +50 daPa. Otoscopic examination was also performed as a way to further ensure normality of the ear-canal and ear drum. All subjects were recruited from a database of potential research subjects that is maintained at Boys Town National Research Hospital. Subjects were paid for their participation. The study described in this article was conducted under an approved Institutional Review Board protocol. After first obtaining informed consent, then audiometric, tympanometric, and otoscopic assessments, data collection was initiated, which required an average of 72 min per subject.

### Measurements

The sound-delivery system consisted of two modified tweeters (TW010F1, Audax, France) acoustically attached by plastic tubes to an ER-10B+ probe microphone (Etymotic Research, Elk Grove Village, IL, USA). Each stimulus condition was repeated on each sound source, and the independent measurements from the two sources were combined into a single average. Amplifiers placed between the soundcard and the tweeters provided power gain and reduced the electrical load on the soundcard output. (The design of the modified tweeters and amplifiers was developed at Northwestern University by J. H. Siegel, who generously shared a prototype with us.) The measurement system was calibrated prior to data collection to determine the Thévenin-equivalent source impedance and pressure (Allen 1986; Keefe et al. 1992; Rasetshwane and Neely 2011).

A WBN signal and a wide-band linear-swept frequency chirp (LSC) signal, both generated digitally at a sampling rate of 48 kHz, were used as stimuli. The duration of each stimulus/response buffer was 171 ms. The WBN stimulus was presented at levels of 20–70 dB SPL in 10-dB steps, and the LSC stimulus was presented at 60 dB SPL. These stimulus levels were determined using a sound level meter (System 824, Larson Davis, Provo, UT, USA) with C weighting, and their range is similar to those routinely used for CEOAE (e.g., Kalluri and Shera 2007) and SFOAE (Choi et al. 2008) measures. The WBN stimulus had constant spectral amplitude and random phase; that is, it was white noise. White noise was chosen for compatibility with the EQ-NL theorem. These stimulus conditions are summarized in Table 2. The fourth column of the table reports the averaging time in seconds. More averaging was done at the lower stimulus levels to mitigate the effect of low signal/noise ratio (SNR) due to the reduced magnitude of the response. Each test condition in Table 2 was repeated four times to further boost the SNR, as described later. The data collected with the LSC stimulus (test 7 in Table 2) are used as a reference signal for estimating CR as described later. Measurements of the response acoustic pressure for the different stimulus conditions were used to determine the ear-canal acoustic impedance from which the reflectance is determined. Stimulus delivery and data collection were controlled using locally developed software (EMAV; Neely and Liu 1994).

**TABLE 2:** Stimulus types and levels

Test	Stimulus	Level (dB SPL)	Averaging time (s)
1	WBN	20	192
2	WBN	30	96
3	WBN	40	48
4	WBN	50	24
5	WBN	60	12
6	WBN	70	6
7	LSC	60	6

Data were collected using wide-band noise (WBN) at levels of 20–70 dB SPL and using a wide-band linear-swept frequency chirp (LSC) at 60 dB SPL. More averaging was done at the lower stimulus levels to mitigate the effect of low SNR. Each test condition was repeated four times for additional SNR boosting. Averaging times are for a single test condition

Reflectance calculations require accurate estimates of the *characteristic impedance* of the ear canal. If the cross-sectional area *A* of an ear canal is known, then the characteristic impedance *Z*_0_ can be calculated using *Z*_0_ = *ρc*/*A*, where *ρ* is the density of air and *c* is the speed of sound. However, it is not possible to measure the cross-sectional area of the ear canal accurately when the OAE probe is inserted in the ear canal. Using the cross-sectional area of the calibration tube is only appropriate if it matches that of the ear canal. Assuming incorrect cross-sectional area, and hence incorrect characteristic impedance, can lead to errors in the calculation of reflectance. Our characteristic impedance estimation procedure avoids the need to know the cross-sectional area of the ear canal by determining the characteristic impedance from the load impedance. The procedure uses an iterative procedure that estimates the “surge” component of the load impedance by minimizing the time-domain ECR and its first derivative at *t* = 0. This method is an improvement to our previous procedure (Rasetshwane and Neely 2011) that only minimized the time-domain ECR at *t* = 0. Surge impedance characterizes the immediate response of an acoustic load to an impulsive stimulus and has been shown to accurately estimate the characteristic impedance of uniform tubes with various diameters (Scheperle et al. 2011). The improved surge estimation procedure provides better estimates of the characteristic impedance of uniform tubes when compared to the previous procedure.

### Estimation of cochlear reflectance

Measurements of ear-canal impedance *Z*_ec_ and estimates of characteristic impedance *Z*_0_ were used to calculate ECR in the frequency-domain using the formula

1 $$ {R_{\text{ec}}}\left( {x,s} \right) = \frac{{{Z_{\text{ec}}}\left( {x,s} \right) - {Z_0}(x)}}{{{Z_{\text{ec}}}\left( {x,s} \right) + {Z_0}(x)}}, $$

where *s* = *i*2*πf* is the Laplace complex frequency variable (e.g., Claerbout 1985). Frequency-domain ECRs were transformed to time-domain ECRs using the inverse Fourier transform. The frequency-domain reflectance was multiplied by a Blackman window with an 18-kHz half-width prior to computation of the time-domain ECR (1) to reduce contribution from higher frequencies, where the measurement was less reliable, and (2) to eliminate ringing in the time domain. A Blackman window has less sideband leakage than equivalent-length Hamming and Hanning windows, which, in this case, reduces spread in the time-domain ECR. The validity of applying a Blackman window to the ECR was recently demonstrated by Rasetshwane and Neely (2011) by showing that the corresponding time-domain representation retains sufficient information to estimate ear-canal shape.

Estimation of CR is done in two stages. In the first stage, we take advantage of the dependence of cochlear reflectance on stimulus level to remove activity due to the middle ear by applying a subtraction procedure. Time–frequency analysis is used in the second stage to remove residual middle-ear activity that was not removed by the subtraction procedure and any measurement-system artifact. Let *r*_WBN, *L*_ (*t*) be the time-domain ECR elicited using WBN (tests 1–6 in Table 2) at stimulus level *L* and let *r*_LSC_ (*t*) be the time-domain ECR elicited using LSC (test 7 in Table 2). Most of the reflected energy in ECRs *r*_WBN, *L*_ (*t*) and *r*_LSC_ (*t*) is linearly related to stimulus intensity and comes from the middle ear; however, a small component is due to reflection from the cochlea. We obtain an estimate of the nonlinear time-domain CR *r*_CR,*L*_ (*t*) at stimulus level *L* as

2 $$ {r_{{C{\text{R,}}L}}}(t) = {r_{{{\text{WBN,}}L}}}(t) - {r_{\text{LSC}}}(t) $$

This subtraction procedure is intended to remove time-domain reflectance contributions that vary linearly with level, especially contributions coming from the middle ear. The LSC stimulus provides an intense excitation in the cochlea that saturates most of the nonlinearities. The WBN stimulus at the highest stimulus intensity also provides intense excitation; however, the LSC stimulus, although changing in time, is more localized at any given instant and was found to produce better estimates of cochlear reflectance in preliminary analysis. The LSC response is thought to have less cochlear contribution than a WBN response at the same level because activity within the cochlea is more localized at any instant in time, due to the instantaneous frequency content of the stimulus, causing local vibration amplitudes to be larger and, therefore, causing contributions from nonlinear elements, which saturate at higher amplitudes, to be relatively smaller. Separate time-domain CRs were computed from the four measurements and averaged to improve the SNR. Additional SNR improvements were obtained by averaging the two time-domain CRs from the two sound sources. An estimate of the noise level was obtained as the difference between repeated measurements.

**FIG. 1.**
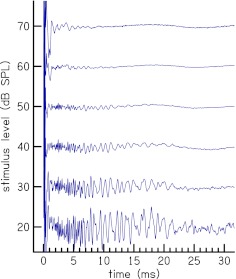
Time-domain cochlear reflectance after the subtraction procedure and before the time–frequency analysis. The *ordinate label* shows the stimulus level and not the level of the time-domain CR. High-frequency content temporally precedes low-frequency content in the time-domain CR. The activity below *t* = 1 ms is middle-ear activity that was not removed by the subtraction procedure.

Figure 1 shows examples of the time-domain reflectances after the subtraction procedure for one subject at stimulus levels of 20–70 dB SPL of WBN stimulus. The ordinate label shows the stimulus level and not the level of the time-domain CR. High frequency content temporally precedes low-frequency content, consistent with signals of cochlea origin. The activity below *t* = 1 ms is residual middle-ear activity that was not removed by the subtraction procedure and measurement-system artifact. This activity is dominant below *t* = 1 because the ear-canal time-domain reflectance has large amplitude for *t* < 1 (see Rasetshwane and Neely 2011, Fig. 5).

To eliminate the residual middle-ear activity and measurement-system artifact time–frequency analysis of CR *r*_CR,*L*_ (*t*) was performed using a complex gammatone filterbank with 49 channels (e.g., Patterson and Holdsworth 1996; Hohmann 2002). The individual gammatone filters of the filterbank were of order *n* = 4 and were implemented using the infinite impulse response algorithm of Härmä (1999). The filterbank was designed such that all the filters had the same tuning of *Q*_ERB_ = 3, with *Q*_ERB_ defined as *f*_c_/ERB (*f*_c_), where *f*_c_ is the center frequency and ERB (*f*_c_) is the equivalent rectangular bandwidth of the filter with center frequency *f*_c_, respectively (see Shera et al. 2010). The center frequencies were logarithmically spaced from *f*_c_ = 0.0625–16 kHz in 1/6-octave steps. The outputs of the gammatone filterbank *r*_CR,*L*_ (*f*_c_, *t*) are complex-valued bandpass-filtered time-domain components of the CR *r*_CR,*L*_ (*t*). The real part of the output of the filterbank represents the band-limited gammatone filter output, whereas the imaginary part approximates its Hilbert transform (Hohmann 2002). The use of complex-valued gammatone filters facilitates accurate calculation of the time-domain envelope since two signals, the imaginary and real parts, are involved in the computation of a single envelope as opposed to use of only one signal when the filter outputs are real. The individual outputs of the gammatone filterbank *r*_CR, *L*_ (*f*_c_,*t*) can experience different delays, which can affect the estimation of the latencies of these outputs. Compensation for delay of the gammatone filters was performed by delaying the fine structure and the envelope of each filter’s impulse response so that all channels have their envelope maximum and their fine-structure maximum at the same time instant, the desired filterbank group delay (Hohmann 2002). The advantage of using the gammatone filterbank over a short-time Fourier transform or a continuous-wavelet transform is that it allows frequency resolution to be specified as desired at both low and high frequencies. Additionally, gammatone filters are often used in psychophysical auditory models (e.g., Patterson and Holdsworth 1996; Meddis et al. 2001; Jepsen et al. 2008) because of their similarity to physiological measures of basilar membrane vibrations (e.g., Rhode and Robles 1974).

**FIG. 2.**
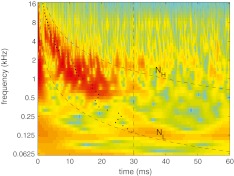
Time–frequency analysis of CR using the gammatone spectrogram. The region of high energy of the gammatone spectrogram enclosed by functions *N*_*L*_ and *N*_*H*_ includes most of the exponential decaying energy of CR. The region below *N*_*L*_ includes residual middle ear and measurement system activity that was not removed by the subtraction procedure. The activity beyond *t* = 30 (indicated with vertical *dashed line*) is due to re-reflection of the traveling wave. The *dots* are estimates of the group delay.

An example of the time–frequency analysis of CR is illustrated in Figure 2 for one of the subjects at a stimulus level of 20 dB SPL, using a spectrogram. We refer to this spectrogram as the *gammatone spectrogram*. In the figure, more reddish color corresponds to larger magnitude compared to more bluish color and the magnitude range (from red to blue) is 108 dB. Also plotted in Figure 2 are functions indicating constant numbers of delay cycles (*N* ≡ *tf*_c_):

3 $$ {N_L} \equiv t{f_{\text{c}}} = 4 + 0.27f $$

4 $$ {N_H} \equiv t{f_{\text{c}}} = 40. $$

The 0.27-ms factor in the definition of *N*_*L*_ [cf. Eq. (3)] is the average round-trip transmission delay to the middle ear ossicles, determined from time-domain ECR (see Rasetshwane and Neely 2011, Fig. 5). The region of high energy of the gammatone spectrogram enclosed by *N*_*L*_ and *N*_*H*_ includes most of the energy of the CR. The region below *N*_*L*_ presumably includes residual middle-ear reflectance and measurement-system artifact that was not removed by the subtraction procedure of Eq. (2). However, at high frequencies (above 8 kHz), the separation of this activity from possible cochlear contributions from the base is not that clear. The activity beyond *t* = 30 ms or above *N*_*H*_ is thought to be due to re-reflection of the traveling wave at the stapes, sound source, and the basilar membrane emission source. We estimate CR in the region enclosed by *N*_*L*_, *N*_*H*_, and *t* = 30 ms. An inspection of all the data collected revealed that the limits *N*_*L*_, *N*_*H*_, and *t* = 30 ms were always a good bound of the exponential decaying cochlear contribution, and the limit *N*_*L*_ was always a reasonable boundary between cochlear reflectance and residual middle-ear activity.

Figure 2 also provides information about CR *latency*. For example, the delay of the center of energy of any given frequency is visualized as the horizontal center of the reddish region along the corresponding vertical location. To estimate the CR latency for a given stimulus level *L*, we compute, for center frequency *f*_c_, the group delay *τ*_CR_ (*f*_c_) of the components *r*_CR,*L*_ (*f*_c_, *t*)

5 $$ {\tau_{\text{CR}}}\left( {{f_{\text{c}}}} \right) = \frac{{\sum\limits_t {t{{\left| {{r_{{{\text{CR}},L}}}\left( {{f_{\text{c}}},t} \right)} \right|}^2}} }}{{\sum\limits_t {{{\left| {{r_{{{\text{CR}},L}}}\left( {{f_{\text{c}}},t} \right)} \right|}^2}} }}. $$

This definition of group delay, which is consistent with Goldstein et al. (1971), corresponds to the time at which the energy of *r*_CR,*L*_ (*f*_c_, *t*) is centered within the spectrogram region described above. The dots in Figure 2 are an example of the group-delay estimates *τ*_CR_ (*f*_c_). The time limits in the summations are defined by the functions *N*_*L*_ and *N*_*H*_:

6 $$ {N_L} < N < {N_H}, $$

and

7 $$ 0 \leqslant t < 30 {\text{ms}}. $$

The time limit *t* = 30 ms is larger than the largest previously reported latency measurement for signals of cochlea origin (see, e.g., Neely et al. 1988) and does not result in an underestimation of *τ*_CR_ (*f*_c_). The time limits of Eqs. (6) and (7) can be interpreted as a windowing method similar to that commonly used for extracting OAEs (see, e.g., Kalluri and Shera 2007), with the added feature that the onset of the window (controlled by *N*_*L*_) varies with frequency. An early onset time is used for high frequencies, allowing us to extract high frequency CR that has short delay, and a late onset time is used for low frequencies to reduce contribution from residual middle-ear reflectance and any measurement-system artifact.

Following Shera and Guinan (2003), we define

8 $$ {N_{\text{CR}}}\left( {{f_{\text{c}}}} \right) \equiv {f_{\text{c}}}{\tau_{\text{CR}}}\left( {{f_{\text{c}}}} \right), $$

a dimensionless group delay by measuring time in cycles or periods of the center frequency of the filters, *f*_c_. A power law fit, i.e., straight-line approximation on the log–log axes, of the form

9 $$ y = a{x^b} $$

can be fit to the group delay data *τ*_CR_(*f*_c_) or the function *N*_CR_(*f*_c_) to characterize the frequency–latency relationship (e.g., Neely et al. 1988). In this equation, parameter *a* is the latency at *f*_c_ = 1 kHz and *x* = *f*_c_/1 kHz.

The estimate of CR *magnitude* (CRM) as a function of frequency is obtained as the root mean square of the outputs of the gammatone filters *r*_CR,*L*_ (*f*_c_, *t*)

10 $$ {\text{CR}}{{\text{M}}_L}\left( {{f_{\text{c}}}} \right) = \gamma \sqrt {{\frac{1}{N}\sum\limits_t {{{\left| {{r_{{{\text{CR,}}L}}}\left( {{f_{\text{c}}}{,}t} \right)} \right|}^2}} }}, $$

where the time limits in the summation are again specified by Eqs. (6) and (7). The scale factor *γ* given by

11 $$ \gamma = \sqrt {{\frac{{2{f_{\text{s}}}}}{{{\text{ERB}}\left( {{f_{\text{c}}}} \right)}}}}, $$

where *f*_s_ is the sampling rate and ERB (*f*_c_) is as defined above, was applied to remove high frequency emphasis (introduced using bandwidths that were proportional to the center frequencies) and to provide for physical interpretation.

**FIG. 3.**
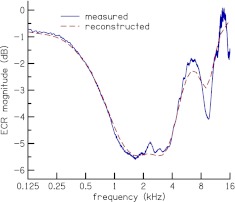
Comparison of average ECR magnitude measured using Fourier transform (*solid line*) to ECR reconstructed from the gammatone-filter outputs (*dashed line*). The reconstructed ECR magnitude is a smooth version of the measured ECR magnitude. The agreement validates the gammatone-filterbank method for computing the CR magnitude [cf. Eq. (10)].

To demonstrate validity of our method for computing the reflectance magnitude [cf. Eq. (10)], Figure 3 compares the average ECR magnitude for the subjects included in this study computed from *r*_LSC_ (*t*) using Eq. (10) to average ECR computed using the magnitude Fourier transform of *r*_LSC_ (*t*). There is agreement between the two methods; the reflectance magnitude obtained with Eq. (10) is a smooth version of the reflectance magnitude obtained as the magnitude Fourier transform. The smoothed spectrum is convenient when comparing spectra across different stimulus levels. The trends in the ECR magnitude of Figure 3 are in agreement with recent ECR measurements reported by Rosowski et al. (2012), which are likewise in agreement with several previous measurements of ECR (e.g., Voss and Allen 1994).

An estimate of CRM as a function of stimulus level is obtained from Eq. (10) by integrating over frequency, i.e.

12 $$ {\text{CR}}{{{\text{M}}}_{L}} = \frac{1}{N}\sum\limits_{{{{f}_{{\text{c}}}}}} {{\text{CR}}{{{\text{M}}}_{L}}} \left( {{{f}_{{\text{c}}}}} \right). $$

where the frequency limits in the summation are again specified by Eqs. (6) and (7).

## Results

**FIG. 4.**
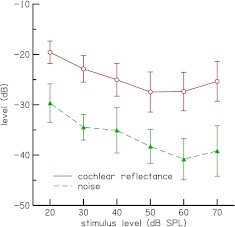
Mean levels ± 1 SD of CR *r*_CR,*L*_ (*t*) (*open circles*) obtained with the subtraction procedure (before time–frequency analysis) and noise floor (*filled triangles*) as functions of stimulus level. The SNR (difference between the two curves) is near constant for stimulus levels of 20–50 dB and increases for higher stimulus levels. The positive SNR demonstrates the reliability of our measurements for all stimulus levels.

Figure 4 shows the mean levels and ±1 standard deviation (SD) of time-domain CR *r*_CR,*L*_ (*t*) after the subtraction procedure, as function of stimulus level for the subjects who participated in this study. The mean noise level, defined as the difference between repeated measurements of time-domain CR, is also shown. The levels of *r*_CR,*L*_ (*t*) are presented as an indicator of the SNR of our data and not as an indicator of the level of the OAE that would be obtained if *r*_CR,*L*_ (*t*) was convolved by the stimulus, since *r*_CR,*L*_ (*t*) include residual contributions from the middle-ear and measurement system. An indicator of the level of the OAE can be obtained from a reflectance that excludes middle-ear activity, as discussed later. The *r*_CR,*L*_ (*t*) SNR is about 10 dB at stimulus levels of 20–50 dB and about 12 dB at higher stimulus levels. The positive SNR demonstrates the reliability of our measurements for all stimulus levels. The SDs for *r*_CR,*L*_ (*t*) describe intersubject variability. Some of this variability is expected from coherent-reflection theory, due to the role of random mechanical irregularity in the process of otoacoustic emission generation (Shera et al. 2008), and some of the variability may be due to measurement noise.

### Cochlear reflectance magnitude

**FIG. 5.**
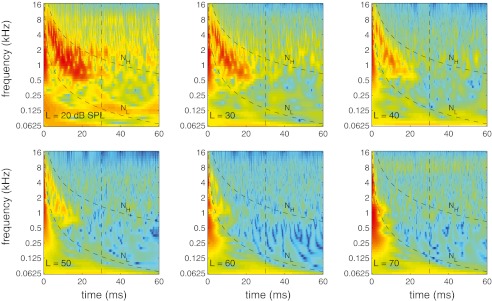
Illustration of CR dependence on stimulus level (*L*) and frequency using gammatone spectrograms. CR can be observed at the four lowest stimulus levels, and its magnitude decreases with increasing stimulus level. There is no evidence of CR at the highest level. CR persists longer at lower stimulus levels than higher levels; there is more re-reflected energy near 30 ms at the lowest level.

Figure 5 shows examples of gammatone spectrograms of CR at six stimulus levels for the same subject whose CR at 20 dB SPL was shown in Figure 2. As stimulus level increases, the energy of the component that we associate with CR (between *N*_*L*_ and *N*_*H*_) decreases and the energy of the component in the region below *N*_*L*_ remains the same or increases; that is why we associate the latter with the middle-ear and the measurement system. The temporal persistence of CR also depends on level; there is more re-reflected energy closer to 30 ms at the lower levels compared to the higher levels.

**FIG. 6.**
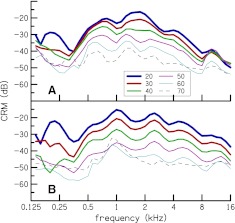
Individual CR magnitude (CRM) as a function of frequency, CRM_*L*_ (*f*_c_) for two subjects, for stimulus levels of 20–70 dB SPL. CRM has spectral oscillations similar to those observed in CEOAE and SFOAE transfer functions by Kalluri and Shera (2007) and in CEOAE transfer functions by Sisto and Moleti (2008). Individual variation between the two subjects can also be observed.

Cochlear reflectance magnitude (CRM) as a function of frequency, CRM_*L*_ (*f*_c_) [cf. Eq. (10)] for two subjects is plotted in Figure 6, for stimulus levels of 20–70 dB SPL. The dependence of CRM on stimulus level is visible—CRM is higher at lower stimulus levels. Individual CRM has spectral oscillations similar to those observed in CEOAE and SFOAE TFs by Kalluri and Shera (2007) and in CEOAE TFs by Sisto and Moleti (2008). However, the notches in the CRM spectra are not as sharp as those in the OAE TFs. Individual variation, in terms of location of peaks and notches, between the two subjects can also be observed.

**FIG. 7.**
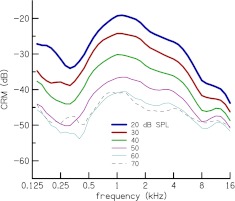
Mean CR magnitude (CRM) as a function of frequency, CRM_*L*_ (*f*_*c*_), for stimulus levels of 20–70 dB SPL. CRM has a bandpass characteristic with energy concentrated between 1 and 2 kHz, at the lower stimulus levels. CRM is larger at lower stimulus levels and saturates at high stimulus levels.

Mean CRM for the entire group of subjects is plotted Figure 7. As expected, averaging over subjects removes the individual spectral oscillations but reveals global spectral features. Mean CRM has a bandpass characteristic with a broad peak centered at 1.1 kHz at stimulus levels of 20–50 dB SPL. The spectral shape of CRM_*L*_ (*f*_c_) becomes flatter at 60 and 70 dB SPL. but the peak at 1.1 kHz remains prominent. This peak, as well as the general spectral shape of our mean CRM, is similar to TEOAE TF of Sisto and Moleti (2008). Kalluri and Shera (2007) also observed this peak in their CEOAE and SFOAE transfer functions.

**FIG. 8.**
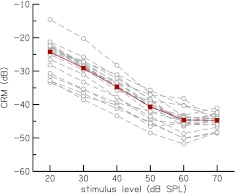
CR magnitude as a function of stimulus level, CRM_*L*_. CRM_*L*_ for individual subjects (*dashed lines with open circles*) and mean for the subjects (*solid line with filled squares*) are shown. Mean CRM_*L*_ has slopes of −0.48 dB/dB at low levels (from *L* = 20 to 30 dB SPL), −0.60 dB/dB at intermediate levels (from *L* = 30 to 60 dB SPL) and −0.06 dB/dB at high levels (from *L* = 60 to 70 dB SPL).

The overall dependence of CRM on level can also be revealed by integrating CRM functions of Figure 7 across frequency to obtain CRM as a function of stimulus level, CRM_*L*_ [cf. Eq. (12)]. Figure 8 shows CRM_*L*_ functions for individual subjects and the group mean CRM_*L*_. Although there is variability in individual subjects, the CRM_*L*_ functions all share the same qualitative form. They all decrease with increases in stimulus level, and most show saturation or onset of saturation at the highest stimulus level, i.e., further increase in stimulus level does not result in considerable decrease in CRM. At low level (from *L* = 20–30 dB SPL) mean CRM_*L*_ has a slope of −0.48 dB/dB. This slope steepens to −0.60 dB/dB at intermediate levels (from *L* = 30–60 dB SPL). At high stimulus levels (from *L* = 60–70 dB SPL), mean CRM_*L*_ becomes independent of level and has a slope near zero (−0.06 dB/dB). The slope of CRM_*L*_ at intermediate levels is similar to the range of slopes, −0.55 to −0.7, of equivalent CEOAE TF analysis of Sisto and Moleti. Kalluri and Shera (2007) obtained a shallower slope of about −0.4 between *L* = 30 and 60 dB SPL in their unified analysis of CEOAE and SFOAE TFs of a single subject, but interestingly as we did, they observed a declining slope (less steep) at low levels. However, their slope at low levels was much shallower than the slope we observed. The reduction in the slope of CRM_*L*_ at low level suggests onset of cochlear linearity.

The level of the OAE at each stimulus level can be deduced by deriving an OAE input/output (I/O) function from the CRM_*L*_ function and it ranges from −5 dB SPL (at *L* = 20 dB SPL) to 26 dB SPL (at *L* = 70 dB SPL). This OAE I/O function (plot not shown) has a slope of 0.40 dB/dB between *L* = 30 and 60 dB SPL and a slope of 0.52 dB/dB between *L* = 20 and 30 dB SPL. The OAE I/O slope of 0.40 is shallower than the mean slopes (0.61 and 0.65) reported by Choi et al. (2008) in their two different analyses of SFOAE I/O functions.

### Reflectance latency

#### Latencies of time-domain components

**FIG. 9.**
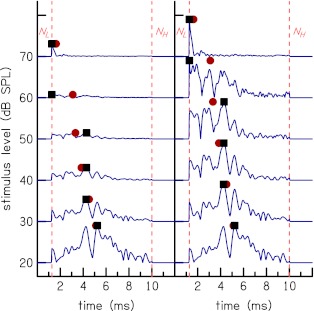
Time-domain CR envelopes at 4 kHz and stimulus levels of 20–70 dB SPL for a single subject. The *ordinate label* shows the stimulus level and not the amplitude of the CR envelopes. CR envelopes on the *right panel* are normalized to the maximum peak for visual clarity. The dashed vertical lines are the limits *N*_*L*_ and *N*_*H*_. Superimposed on both panels are the envelope delay (*filled squares*) and group delay, *τ*_CR_ (*filled circles*). Peaks persist across level, and the amplitudes of the peaks are largest at the lowest level.

An example of the time-domain CR envelope is shown in Figure 9 for one subject for center frequency *f*_c_ = 4 kHz and stimulus levels of 20–70 dB SPL. This analysis shows the variation in the envelope of the time-domain CR as a function of stimulus level at a given center frequency. In terms of the function *r*_CR, L_ (*f*_c_, *t*), Figure 9 shows ${\left. {{r_{{{\text{CR}},L}}}\left( {{f_{\text{c}}} = 4,t} \right)} \right|_{{L = 20,30,...70{\text{dB}}}}}$. The CR envelopes displayed in the right panel are normalized to the maximum peak for visual clarity, and the envelopes displayed in the left panel are not normalized to show the dependence of their amplitude on stimulus level. The dashed vertical lines are the limits *N*_*L*_ and *N*_*H*_ [cf. Eqs. (3) and (4)]. Superimposed on the plots are the delay of the dominant peak or envelope delay (filled squares) and group delay, *τ*_CR_ (*f*_c_ = 4) (filled circles). It is interesting to note that the CR envelopes have multiple peaks at each level, not just the dominant peak and the timings of these peaks persist across level. The amplitudes of the peaks are large at low stimulus levels and decrease with increases in stimulus levels. Recall that a similar trend (decrease in CR with increase in stimulus level) was observed for the functions CRM_*L*_ (*f*_c_) and CRM_*L*_. The peak at *t* = 1.3 ms is the dominant peak at *L* = 60 and 70 dB SPL. The peak at *t* = 4.2 ms, which is small at *L* = 60 and 70 dB SPL, becomes the dominant peak from *L* = 50 to 30 dB SPL. That is, the dominant peak, as well as the group delay, shifts to later times as the stimulus level decreases.

**FIG. 10.**
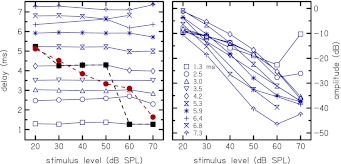
Latency (*left panel*) and amplitude (*right panel*) of peaks shown in Fig. 9, as functions of stimulus level. Superimposed on the *left panel* are the envelope delay (*filled squares*) and group delay, *τ*_CR_ (*filled circles*). Only peaks with delays of up to the maximum of the envelope delay plus 2 ms are shown, to reduce clutter in the plot. Delay peaks persist across level. The amplitude of the peaks decrease with increasing stimulus level, with later occurring peaks having amplitudes with a wider dynamic range compared to earlier occurring peaks.

The latencies and non-normalized amplitudes of the CR envelopes of Figure 9 are depicted in Figure 10 as functions of stimulus level. Only peaks with delays of up to the maximum of the envelope delay plus 2 ms are shown, to reduce clutter in the plot. Several features are noteworthy. The peaks at a given delay persist across stimulus level (see left panel), with only minor shifts in their timings at the higher stimulus levels. Superimposed on this panel are the envelope delay (filled squares) and group delay, *τ*_CR_ (*f*_c_ = 4) (filled circles). At *L* = 20 dB SPL, the peak with a delay of 5.3 ms (hour-glass symbol) is the dominant peak. From *L* = 30 to 50 dB SPL, the peak with a delay of 4.2 ms (diamond symbol) is the dominant peak, and at *L* = 60 and 70 dB SPL, the peak with a delay of 1.3 ms (square symbol) is now the dominant peak. The group delay, like the envelope delay, decreases with increasing stimulus level, but assumes different values as explained later.

Another interesting observation is that the amplitude of the envelope peaks decrease with increases in stimulus level and that the dynamic range of the amplitude of earlier peaks is less than the dynamic range of the amplitude of later peaks. For example, the amplitude of the peak with a delay of 1.3 ms (square symbol) is −10.3 dB (largest value) at *L* = 70 dB and −22.6 dB (smallest value) at *L* = 60 dB, a dynamic range of 12.3 dB. In comparison, peaks with delays of 6.8 ms (picnic-table symbol) and 7.3 ms (upward-arrow symbol) have amplitudes with dynamic ranges of 33.3 and 35.8 dB, respectively. In addition to having a wider dynamic range, the amplitudes of the later occurring delay peaks also have a lower threshold compared to the earlier occurring delay peaks; that is, low level stimulation produces large amplitude. The shift in envelope delay with increasing stimulus levels can also be observed in the right panel. At *L* = 20 dB SPL, the peak with a delay of 5.3 ms (hour-glass symbol) has the largest amplitude. As the stimulus level increases (at *L* = 30 to 50 dB), the peak with a delay of 4.2 ms (diamond symbol) has the largest amplitude, and at *L* = 60 and 70 dB SPL, the peak with a delay of 1.3 ms (square symbol) has the largest amplitude.

**FIG. 11.**
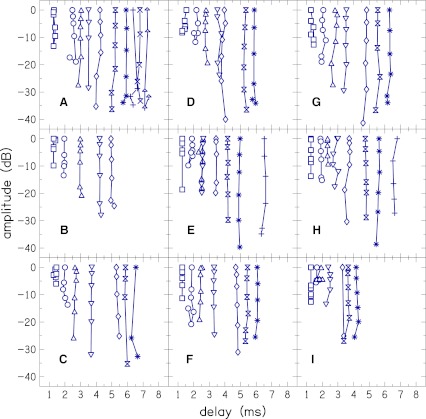
Level series analysis for nine subjects. Stimulus level increases from *top to bottom* and the *length of the lines* indicates amplitude growth. The following observations can be generalized to the subjects (1) delay peaks persist across level, (2) amplitude of the peaks decrease with increasing stimulus level (movement from top to bottom), and (3) later occurring peaks have amplitudes with a wider dynamic range than the earlier occurring peaks.

An alternative analysis of the level series of the CR envelopes of Figure 10 is presented in Figure 11A. For each level series, responses to the lowest stimulus level were normalized to 0 dB. Therefore, for each level series, stimulus level increases from the top to the bottom in the figure. The length of the lines indicates amplitude growth. The following features that were observed in Figure 10 are also evident in this figure: (1) persistence of delay peak across level, (2) decrease in amplitude peaks with increasing level, and (3) amplitude peaks with a wider dynamic range for the later occurring delay peaks.

Figure 11 also extends the level series analysis to eight other subjects. The qualitative analysis is consistent across subjects, although individual variability, more especially in the timings of the delay peaks, exists among subjects. The highly individual timings of the delay peaks and total number of peaks in a given time interval makes averaging results across subjects difficult. However, an analysis that averages the individual group delays is possible and is used to derive the latency–frequency relation.

#### Latency–frequency relation

**FIG. 12.**
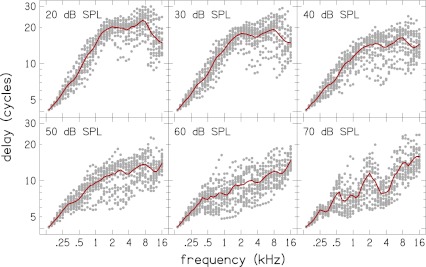
*N*_CR_ versus frequency at different stimulus levels. The group delay *N*_CR_ at each center frequency for all the subjects (*dots*) are plotted together for a given stimulus level, together with Loess trend lines to guide the eye. A breakpoint frequency *f*_*a*|*b*_ can be determined by inspection of the Loess trend lines.

The dependence of latency on frequency and stimulus level is evaluated by considering scatter plots of our estimates of group delay in the dimensionless quantity *N*_CR_ [cf. Eq. (8)], at different stimulus levels in Figure 12. Loess trend lines (Cleveland 1979) are also shown in the figure (solid lines) to guide the eye. A systematic variation in group delay with frequency can be visualized. Except for stimulus levels of 60 and 70 dB SPL, a breakpoint frequency, *f*_*a*|*b*_ can be observed. Below this frequency, *N*_CR_ increases with increasing frequency and above, *N*_CR_ is near independent of frequency. The average value for the break point frequency is *f*_*a*|*b*_ = 1.5 kHz. This value was determined by finding the intersection of power-law fits to the steady-state low and high frequency portions of each latency–frequency curve. The breakpoint, or transition, frequency has been suggested to represent a transition between a high frequency region (*f* > *f*_*a*|*b*_) of more “basal-like” cochlea behavior and a low-frequency region (*f* < *f*_*a*|*b*_) of more “apical-like” cochlea behavior (Shera et al. 2010).

**FIG. 13.**
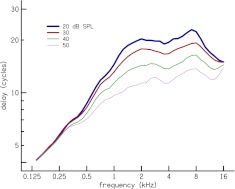
Latency–frequency relation at stimulus levels of 20–50 dB SPL. The Loess trend lines for the latency functions *N*_CR_ at different stimuli levels are plotted together. Above the breakpoint frequency, *f*_*a*|*b*_ = 1.5 kHz, *N*_CR_ has a near zero slope. The slopes of *N*_CR_ below and above *f*_*a*|*b*_ are presented in Table 3. CR delay is longer at low levels, compared to high levels. The latency estimates merge below 0.4 kHz, where there is uncertainty about the estimates.

**FIG. 14.**
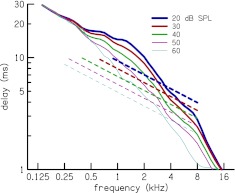
Comparison of cochlear reflectance delay in milliseconds (*solid lines*) to tone-burst ABR forward latency (*dashed lines*) at stimulus levels of 20–60 dB SPL. The decrease in CR latency per decibel increase in level is similar to that of ABR forward latency between 2 and 8 kHz.

The Loess trend lines for the latency–frequency functions *N*_CR_ in the different panels of Figure 12, except for *L* = 60 and 70 dB SPL, are superimposed in Figure 13. The dependence of latency on stimulus level can be observed. For a given frequency, the latency is shorter at higher stimulus levels compared to lower levels. The slope of *N*_CR_ above *f*_*a*|*b*_, but below 8 kHz, is near zero at all stimulus levels and the slope below *f*_*a*|*b*_ ranges from 0.42 to 0.74. There is an orderly decrease in slope below *f*_*a*|*b*_ with increase in level. These slopes, determined via power law fits to the *N*_CR_ trend lines, are presented in Table 3. The latency functions of Figure 13 merge below 0.4 kHz where there is uncertainty in the latency estimation due a reduction in CR energy at low frequencies (cf. Fig. 2). The latency functions also converge at 16 kHz, which is probably influenced by the short time interval between the limits *N*_*L*_ and *N*_*H*_ at high frequencies. An equivalent plot of CR latency in millisecond versus frequency *τ*_CR_(*f*_c_) is shown in Figure 14.

**TABLE 3:** Slopes of the group delay function *N*_CR_ below and above the breakpoint frequency *f*_*a*|*b*_ = 1.5 kHz

Stimulus level (dB SPL)	Slope below *f*_*a*|*b*_	Slope above *f*_*a*|*b*_
20	0.74	0.05
30	0.65	0.02
40	0.55	0.04
50	0.42	0.09

The slope of *N*_CR_ was determine using data only up to 8 kHz. The slope of *N*_CR_ above *f*_*a*|*b*_ is near zero. There seems to be an orderly decrease in slope below *f*_*a*|*b*_ with increase in level. The slopes were determined via power law fits to the *N*_CR_ loess trend curves

## Discussion

A method has been developed for obtaining CR from WBN-ECR measurements. CR constitutes mainly the nonlinear component of ECR and is equivalent to a measure of OAE normalized by the stimulus. The method includes two stages: (1) a subtraction procedure that removes middle-ear reflectance and (2) time–frequency analysis to remove residual middle-ear reflectance and measurement-system artifact. Analyses were performed to examine the level dependence of latencies of individual peaks of time-domain CR and to examine the global dependence of CR on frequency and level. Use of noise to evoke OAEs has previously been done by Maat et al. (2000). However, the purpose of their study was to demonstrate that noise-evoked OAEs are similar to CEOAEs, and as such, their noise stimulus was band-limited to 0.8–5 kHz (which is less than the bandwidth of our CR measurements) to match the spectrum of a click stimulus they used to evoke CEOAE. To the authors’ knowledge, this is the only other study where noise was used to evoke OAEs.

In plots of time-domain CR obtaining using the subtraction procedure (cf. Fig. 1), high frequency-content temporally precedes low-frequency content, which is consistent with signals of cochlea origin. These time-domain CR plots are similar to time-domain plots of CEOAE (e.g., Maat et al. 2000; Avan et al. 2000). Our CR though has a wider bandwidth, when compared to CEOAEs measured in humans using the commonly used nonlinear residual method, which discards the short latency part of the response (e.g., Kemp et al. 1986). However, the double-evoked CEOAE paradigm described by Keefe et al. (2011) has a similarly wide bandwidth of up to 16 kHz. The wide bandwidth of our time-domain CR allows for measurement of CR magnitude and latency that represent more than 80 % of the length of the cochlea (Greenwood 1990).

Gammatone filterbanks were used in the time–frequency analysis to separate CR from residual middle-ear reflectance and measurement-system artifact. A gammatone filterbank has not been used for OAE analysis in previous studies. Other time–frequency analysis techniques, such as the short-time Fourier transform and wavelet transform, may also be used instead of the gammatone filterbank and will produce similar results. The gammatone filterbank, like most other filters, introduces dispersion to the reflectance; however, this effect is small.

The limits *N*_*L*_ and *N*_*H*_ [cf. Eqs. (3) and (4)] are important for separating middle-ear activity from cochlea activity, and their settings were carefully selected based on inspection of a large number of gammatone spectrograms. These limits do not bias the estimation of latency significantly because an inspection of a large number of gammatone spectrograms showed that most of the CR is included within these limits. The residual activity in the CR before application of time–frequency is probably due to variations in time caused by shifts in the position of the measurement probe between measurements. The limit *N*_*L*_ provides a good separation between cochlear contributions and middle-ear and measurement-system activity, except maybe at high frequencies (above 8 kHz), where there are possible short delay contributions from the cochlear base. This ambiguity in the separation of cochlear reflectance and residual activity may have been responsible for the convergence observed in the CR latency estimates at 16 kHz.

The notches in the CRM of Figure 6 are not as sharp as notches that have been observed in magnitude spectra of OAEs and transfer functions of OAEs (e.g., Kalluri and Shera 2007; Norton and Neely 1987; Sisto and Moleti 2008) because our reflectance magnitude computation method [cf. Eq. (10)] smoothes out the sharpness of the notches, as demonstrated in Figure 3. However, the smoothed spectrum still reveals the dependence of CRM on stimulus level and frequency and facilitates comparison of spectra across different stimulus levels. The sharpness of the notches of CRM can be increased either by decreasing the bandwidth of the filters in the filterbank analysis or using a time–frequency analysis method that offers perfect reconstruction, such as wavelets and the short-time Fourier transform. Mean CRM as a function of frequency (cf. Fig. 7) has a peak at 1.1 kHz that has been observed in previous OAE measurements (e.g., Sisto and Moleti 2008). This peak is probably the effect of round-trip transmission through the middle ear as was previously demonstrated by Puria (2003) and Sisto and Moleti (2008).

CRM as a function of stimulus level has a constant slope of −0.6 dB/dB for intermediate levels and saturates at high levels (cf. Fig. 8). Gorga et al. (2011) observed a slope of about −0.6 dB/dB in their estimation of cochlear-amplifier gain using tip-to-tail differences of DPOAE suppression tuning curves. This similarity suggests that CRM decreases with increase in level because the cochlear-amplifier gain decreases. CRM saturates at the higher stimulus levels and becomes independent of stimulus level because at these levels the cochlear-amplifier is not as active (e.g., Kemp 2002; Harte et al. 2009). The acoustic reflex, which causes impedance mismatch in the middle ear and decreases the transmission of energy to the cochlea, may also be responsible for the reduction in CRM at the higher levels. At low levels, the slope of the CRM becomes shallower (−0.48 dB/dB), suggesting onset of cochlear linearity. Additional measurements at levels below 20 dB SPL are required to determine the low-level linear range of CR.

The level-series analysis of time-domain components of CR (cf. Figs. 9, 10 and 11) revealed that (1) delay peaks persist across level with timings unchanged, (2) amplitude of the peaks decrease with increasing stimulus level, and (3) later occurring peaks have amplitudes with a wider dynamic range than earlier occurring peaks. The persistence of the timings of the peaks can also be noted in Figure 1. Our last two observations may seem to contradict those of Stover et al. (1996) and Goodman et al. (2011). In their temporal analysis of DPOAE, Stover et al. (1996) noted that the amplitudes of early occurring peaks had a wider dynamic range than those of later occurring peaks and that amplitude of peaks grow with stimulus level. Goodman et al. (2011) made similar observations in their time-domain analysis of TEOAE. However, CR is a form of an otoacoustic emission that is normalized by the stimulus. When this is taken into consideration, our observations are consistent with these earlier observations. We believe that the multiple delay peaks do not signify multiple sources of OAE generation along the BM, but are due to interference and re-reflections of delayed signals coming back from various points along the BM.

In the level-series analysis and in general, the group delay is different from the envelope delay because the group delay represents a weighted average delay of all the peaks, not just the dominant peak. However, at the lower stimulus levels where the amplitude of the dominant peak is much greater than the amplitude of the other peaks, the group delay and envelope delay are similar. The similarities of the results of our level series analysis to previous results indicate that our measurements are indeed of cochlear origin.

Our measurements of CR can be considered as a superposition of multiple SFOAE measurements that have been normalized by the stimulus. In terms of the two OAE generator mechanisms, CR can then be considered to be due to linear coherent reflection. However, an important difference between the CR and SFOAE exists. SFOAE is typically measured using a pure tone at a single frequency, which, at low stimulus levels, excites a spatially limited region of the cochlea. On the other hand, the wideband stimulus used to measure CR simultaneously excites an extensive region of the cochlea, perhaps the entire cochlea, at the same time. This brings up possibilities for intermodulation products generated by interaction between different frequency components of the stimulus, as was observed by Yates and Withnell (1999) for TEOAEs. Although this complicates the comparison of our measurements to current measures of OAE, the advantage of using a wideband noise stimulus is that it allows for the invocation of the EQ-NL theorem (de Boer 1997), which simplifies the interpretation of CR. The EQ-NL theorem allows the nonlinear mechanics of the cochlea to be replaced by an equivalent linear system for each stimulus level. Linear systems are easier to analyze than nonlinear systems because most analysis tools (e.g., transfer function, Fourier analysis) assume linearity.

The dependence of CR latency on frequency and level is consistent with earlier estimates of OAE and auditory brainstem response (ABR) latency (e.g., Neely et al. 1988; Harte et al. 2009). A comparison of CR latency to tone-burst ABR forward latency of Neely and Gorga (1998) is shown in Figure 14. The decrease in ABR forward latency per decibel increase in level is 1.62 %, at all levels and frequencies. In the current measurements, CR latency between 2 and 8 kHz and for level below 50 dB SPL decreases by a mean value of 1.65 % per dB increase in level, giving further support to our measurements being of cochlear origin. However, the fact that CR latency is not proportional to twice the ABR forward latency (except at approximately 1 kHz) brings into question the prevailing interpretation of OAE latency as being twice the travel time of a particular frequency component from stimulus onset through the cochlea to its characteristic place. Another distinct feature of ABR latencies is that they do not have any apparent “breakpoint frequency” as has been observed for CR and other OAE latencies. We do not yet have clear answers to these questions.

The observed breakpoint in the latency (cf. Fig. 12) is also consistent with earlier studies of OAEs in humans and other mammals (Shera and Guinan 2003; Shera et al. 2010; Schairer et al. 2006; Bergevin et al. 2008; Dhar et al. 2011). Our breakpoint frequency of 1.5 kHz agrees exactly with recent estimates by Dhar et al. (2011) and Abdala and Dhar (2012) using DPOAE. Although there are difference in the breakpoint frequencies compared by the other investigators, the breakpoint frequencies all occurs near the mid-point of the cochlea (Shera et al. 2010). In the current study, the observed breakpoint of 1.5 kHz maps to a distance of 1.8 cm from the stapes according to the Greenwood frequency–place map (Greenwood 1990), which is the midpoint since a typical length of a human cochlea is about 3.5 cm. The breakpoint, or transition, frequency has been suggested to represent a transition between a high-frequency region (*f* > *f*_*a*|*b*_) of more “basal-like” cochlea behavior and a low-frequency region (*f* < *f*_*a*|*b*_) of more “apical-like” cochlea behavior (Shera et al. 2010). For frequencies above the breakpoint *f*_*a*|*b*_, the slope of the latency–frequency function *N*_CR_ is near zero, suggesting that the cochlea has scaling symmetry (Shera et al. 2000). However, scaling symmetry may still hold below the breakpoint frequency, despite a non-zero slope in the delay if the reflection occurred at a basal tail of a cochlear travelling wave with a scaling-symmetric excitation pattern (Choi et al. 2008).

The slopes of the latency–frequency function below the breakpoint frequency decreases with increasing stimulus level (cf. Fig. 13 and Table 3). This decline in latency slope with increasing level was also noted by Sisto and Moleti (2007) for TEOAEs. However, it is possible that the merging of the latency functions below 0.4 kHz may have influenced the observed decline in latency slope, and perhaps, the slopes at the different levels are actually similar.

## Conclusion

CR measured in this study has features that are characteristic of signals of cochlear origin. The CR measurements offer three advantages over other types of otoacoustic emissions: (1) wider bandwidth, (2) minimal influence from the measurement system and middle-ear on the recorded emission, and (3) easier interpretation due to the existence of an equivalent linear model, which validates the application of linear-systems theory. CR provides an innovative and potentially informative means for assessing cochlear processing, compared to other noninvasive examinations of the human cochlea.
